# Comparison of severe hyponatremia in patients with and without psychiatric diseases: A single‐center retrospective study

**DOI:** 10.1002/pcn5.77

**Published:** 2023-01-26

**Authors:** Eriko Makino, Takahide Hashimoto, Akahito Sako, Hideki Nanasawa, Tetsuro Enomoto, Tatsuro Hayakawa, Hidetaka Hamasaki, Hidekatsu Yanai

**Affiliations:** ^1^ Department of Internal Medicine, Kohnodai Hospital National Center for Global Health and Medicine Ichikawa Chiba Japan; ^2^ Department of Psychiatry, Kohnodai Hospital National Center for Global Health and Medicine Ichikawa Chiba Japan

**Keywords:** antipsychotics, polydipsia, schizophrenia, SIADH

## Abstract

**Aims:**

Hyponatremia is a common electrolyte disorder. The severe hyponatremia has a mortality rate of 4%–40%. Psychiatric patients are likely to develop the condition because of polydipsia or the adverse effects of antipsychotics. We investigated the characteristics of patients with and without psychiatric diseases who developed severe hyponatremia.

**Materials and Methods:**

We retrospectively investigated cases admitted to our hospital (all departments) between October 2012 and November 2015 with a serum sodium concentration of ≤125 mmol/l on admission. We compared patient characteristics, etiology, and clinical course between psychiatric and nonpsychiatric patients.

**Results:**

In total, 123 cases (62 female) were analyzed. Psychiatric disorders were present in 69 cases (56%), including schizophrenia (*n* = 19), anorexia (*n* = 16), mood disorders (*n* = 14), and organic mental disorders (*n* = 9). The mean patient age was 63 years. The mean serum sodium concentration on admission was 119 mmol/l, and the main causes of hyponatremia were polydipsia (20%), insufficient sodium intake (18%), and syndrome of inappropriate antidiuretic hormone secretion (16%). Compared with the nonpsychiatric group, the psychiatric group was significantly younger (55 vs. 74 years), was more likely to have polydipsia (30% vs. 6%), and had a lower in‐hospital mortality (0% vs. 17%).

**Conclusions:**

Our study found differences in the clinical picture between psychiatric and nonpsychiatric patients with severe hyponatremia. Clinicians need to monitor serum sodium because the symptoms of hyponatremia can mimic those of psychiatric diseases.

## Graphical abstract

We analyzed 123 severe hyponatremia inpatients (62 female, 69 psychiatric cases). The main causes of hyponatremia were polydipsia (20%), insufficient sodium intake (18%), and syndrome of inappropriate antidiuretic hormone secretion (16%). Compared with the nonpsychiatric group, the psychiatric group was significantly younger (55 vs. 74 years), was more likely to have polydipsia (30% vs. 6%), and had a lower in‐hospital mortality (0% vs. 17%).

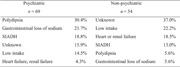

## INTRODUCTION

Hyponatremia is a common electrolyte disorder.^1^ Although large‐scale data in Japan are lacking, its economic burden is substantial and the estimated direct costs of hyponatremia treatment in the United States range from $1.6 to $3.6 billion annually.^2^ Analyses of patients in large hospitalization databases in the United States show that hyponatremia is associated with a 7.6% increase in hospital length of stay and an 8.9% increase in hospital costs as well as increased risk of intensive care unit admission and 30‐day hospital readmission for hyponatremia.^3^

Psychiatric patients are likely to develop hyponatremia because it can occur either due to polydipsia or as an adverse effect of antipsychotics and antidepressants.^4^, ^5^, ^6^, ^7^, ^8^ Previous Japanese research showed that 10.5% of inpatients with psychiatric diseases had hyponatremia.^9^ An American study in a psychiatric hospital found a 6.49% prevalence of hyponatremia (sodium level <136 mEq/l) on admission.^10^ It is not easy to diagnose hyponatremia because the symptoms, such as malaise and appetite loss caused by hyponatremia, are nonspecific and mimic those of psychiatric diseases. Although the cut‐off value of severe hyponatremia, clinical setting, and patient characteristics differed among previous studies, the mortality rate of severe hyponatremia ranged from 4% to 40%.^11^, ^12^, ^13^, ^14^, ^15^ Nonetheless, the clinical data on hyponatremia in psychiatric patients, especially in comparison with nonpsychiatric patients, are lacking.

In this study, we investigated the characteristics, etiology, and clinical course of patients with and without psychiatric disorder who developed severe hyponatremia.

## MATERIALS AND METHODS

This single‐center retrospective observational study was conducted at Kohnodai Hospital, which is an acute care general hospital close to Tokyo. It has over 300 inpatients beds, about half of which are for psychiatric patients because it was formerly the National Center for Neurology and Psychiatry.

Severe hyponatremia was defined as a serum sodium concentration ≤125 mmol/l based on previous studies.^14^, ^16^ Cases of severe hyponatremia on admission between October 2012 and November 2015 were extracted from a clinical database. Cases in which patients with severe hyponatremia who were not hospitalized or who developed hyponatremia after admission were excluded.

We reviewed the inpatient medical records and collected clinical data such as age, sex, main diagnosis for admission, laboratory data, hyponatremia cause, comorbidities, underlying psychiatric diseases, medication, and clinical outcomes. The cause of hyponatremia was determined and registered by the physician in charge and we did not correct it retrospectively.

Psychiatric and nonpsychiatric cases were compared using the *χ* ^2^ test, Fisher's exact test, and *t*‐test as appropriate. Patients using hypnotics for insomnia were not considered psychiatric cases. Variables are expressed as mean ± standard deviation. *P* < 0.05 was considered significant and IBM SPSS Statistics 22 was used for the statistical analyses. The institutional review board approved the study protocol (NCGM‐G‐001911).

## RESULTS

**Figure 1:**
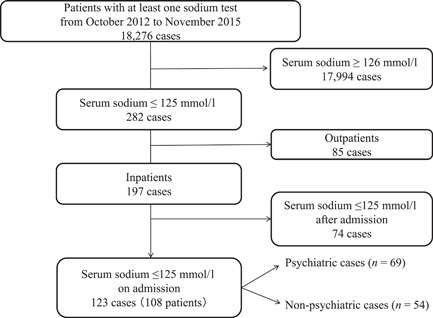
Patient flow diagram.

During the study period, 18,276 cases had at least one blood test for serum sodium. Among them, there were 123 cases with severe hyponatremia on admission (Figure 1).

### Patient demographic data

Sixty‐nine cases involved underlying psychiatric diseases. The most common psychiatric disorders were schizophrenia (19 cases), followed by eating disorders (16 cases), mood disorders (14 cases), organic mental disorders (nine cases), and others. Nonpsychiatric patients were significantly older and had more comorbidities, including malignancies, heart failure, and respiratory diseases. Serum sodium levels were similar but the in‐hospital mortality rate was significantly higher in nonpsychiatric patients than in psychiatric patients (Table 1). The main diagnoses for hospitalization were hyponatremia in 32 cases, infectious diseases (19 cases), anorexia nervosa (15 cases), psychiatric diseases (nine cases), heart diseases (eight cases), liver diseases (seven cases), and gastroenterological diseases (four cases). The proportion of hyponatremia in the main diagnosis for hospitalization was 27.5% in psychiatric patients and 24.1% in nonpsychiatric patients.

**Table 1:** Patient characteristics.

	Psychiatric	Nonpsychiatric	*P* value
	*n* = 69	*n* = 54	
Female sex (%)	54%	44%	0.31
Age (years)	55.3 ± 20.1	73.6 ± 11.4	<0.01
Height (cm)	157.4 ± 9.0	154.0 ± 11.0	0.13
Weight (kg)	50.4 ± 20.1	51.9 ± 11.8	0.67
Serum sodium (mEq/l)	120.0 ± 4.7	118.5 ± 15.6	0.47
Serum creatinine (IU/l)	0.99 ± 0.97	1.02 ± 0.91	0.85
Serum creatinine kinase (IU/l)	1615 ± 8107	442 ± 916	0.30
Serum osmolarity (mOsm/l)	252.6 ± 22.7	249.5 ± 13.6	0.58
Urine sodium (mEq/l)	77.2 ± 51.2	64.5 ± 32.4	0.29
Urine osmolarity (mOsm/l)	299.3 ± 167.3	423.5 ± 160.0	0.02
Comorbidities			
Malignancies	0.0%	27.8%	<0.01
Diabetes	18.8%	16.7%	0.82
Renal failure	4.3%	13.0%	0.10
Respiratory diseases	17.4%	40.7%	<0.01
Heart failure	2.9%	13.0%	0.04
Liver cirrhosis	4.3%	13.0%	0.10
Central nervous system diseases	31.9%	22.2%	0.31
Medications			
Any psychotropic drugs	79.7%	25.9%	<0.01
Antipsychotics	47.8%	1.9%	<0.01
Antidepressants	27.5%	0.0%	<0.01
Benzodiazepines	58.0%	18.5%	<0.01
Mood stabilizers or antiseizure drugs	18.8%	5.6%	0.03
Diuretics	10.1%	27.8%	0.02
Angiotensin II receptor blockers	14.5%	29.6%	0.05
Proton pump inhibitors	33.3%	35.2%	0.85
Nonsteroidal anti‐inflammatory drugs	15.9%	14.8%	1.00
Length of stay (days)	28.2 ± 27.8	25.4 ± 22.6	0.55
In‐hospital mortality (%)	0.0%	16.7%	<0.01

### Hyponatremia etiology

Polydipsia was the leading cause of hyponatremia in psychiatric patients, followed by gastrointestinal loss of sodium and syndrome of inappropriate antidiuretic hormone secretion (SIADH) (Table 2). Among the 15 cases with gastrointestinal loss, there were 14 with an eating disorder and behavior of self‐induced vomiting and abuse of laxatives. In contrast, insufficient oral intake was the leading cause of hyponatremia in nonpsychiatric cases, followed by renal and/or heart failure and SIADH. The etiology of hyponatremia was not determined in 25% of all cases (37% of nonpsychiatric cases and 16% of psychiatric cases). Laboratory tests were performed at similar rates in the two groups as follows: urine sodium in 44%, serum osmolality in 40%, and urine osmolality in 37%.

**Table 2:** Hyponatremia etiology.

Psychiatric		Nonpsychiatric	
*n* = 69		*n* = 54	
Polydipsia	30.4%	Unknown	37.0%
Gastrointestinal loss of sodium	21.7%	Low intake	22.2%
SIADH	18.8%	Heart or renal failure	18.5%
Unknown	15.9%	SIADH	13.0%
Low intake	14.5%	Polydipsia	5.6%
Heart failure, renal failure	4.3%	Gastrointestinal loss of sodium	5.6%

Abbreviation: SIADH, syndrome of inappropriate secretion of antidiuretic hormone.

There were 20 cases of SIADH. The mean age was 75 ± 7 years and 45% were women. Serum sodium was 121 ± 4 mmol/l, urine osmolarity was 398 ± 159 mOsm/l, and urine sodium was 88 ± 49 mmol/l. Serum creatinine kinase was 684 ± 2108 IU/l. Of these, 65% were cases with psychiatric diseases. In terms of the etiology of SIADH, 10 cases were drug‐induced (six antidepressants, two antipsychotics, one antiseizure drug, and one supplement), five had lung diseases (two lung cancer, one pneumothorax, one pyothorax, and one hypersensitivity pneumonitis), two had central nervous system disorders (subdural hemorrhage and chronic subdural hematoma), and three did not have a clear etiology. None of these patients died during hospitalization.

There were 24 cases of polydipsia. The mean age was 52 ± 16 years and 38% were women. Serum sodium was 117 ± 5 mmol/l, urine osmolarity was 191 ± 110 mOsm/l, and urine sodium was 38 ± 19 mmol/l. Serum creatinine kinase was 1615 ± 8107 IU/l. Eighty‐seven percent were cases with psychiatric diseases (50% had schizophrenia) and 70% were taking antipsychotics. None of these patients died during hospitalization.

### Clinical outcomes and characteristics of nonsurvivors

None of the enrolled patients developed osmotic demyelination syndrome (formerly called central pontine myelinolysis) caused by overly rapid correction of hyponatremia. Nine patients died during hospitalization and all were in the nonpsychiatric group. In the nonsurvivor group, the mean age was 65 years and the mean serum sodium concentration on admission was 119 mmol/l. The main causes of death were not hyponatremia, but rather breast cancer, liver cirrhosis, and heart failure in two patients each and peritonitis, Evans syndrome, and hepatocellular carcinoma in one patient each.

## DISCUSSION

Our retrospective single‐center study revealed differences in the patient characteristics, etiology, and mortality of hyponatremia in patients with and without psychiatric diseases. Patients with psychiatric diseases were younger and had fewer underlying diseases. Polydipsia, gastrointestinal loss of sodium due to an eating disorder, and SIADH were common causes of hyponatremia in psychiatric patients. Although serum sodium levels on admission were similar in the two groups, the nonpsychiatric group had higher mortality. To our knowledge, no previous studies have investigated patients with hyponatremia and psychiatric diseases and elucidated the differences from nonpsychiatric patients.

Psychiatric patients tend to develop more severe physical diseases and to have worse clinical outcomes compared with nonpsychiatric patients.^17^, ^18^ Life expectancy is shorter in individuals with a mental disorder than in the general population, and this is due more to natural death, including cardiovascular diseases and infections, than to suicide.^19^, ^20^ This difference may be explained by lower accessibility to physical healthcare resources and difficulty describing their symptoms compared with the general or nonpsychiatric population. In the present study, serum sodium levels on admission were similar in both psychiatric and nonpsychiatric patients. Moreover, mortality was lower in psychiatric cases than in nonpsychiatric cases. Because of the clinical setting, a general hospital, it is easier to frequently monitor serum sodium levels and manage hyponatremia in collaboration with internists than in a psychiatric specialized hospital and clinic. The older age and higher number of underlying diseases in the nonpsychiatric patients might outweigh the burden of mental diseases.

The etiology of the hyponatremia in this study differed between patients with psychiatric diseases and those without. The leading cause of hyponatremia in two academic centers in Switzerland was primary polydipsia, followed by diuretic‐induced hyponatremia and SIADH.^15^ A multicenter study in the UK found that SIADH was the leading cause of hyponatremia, followed by gastrointestinal loss of sodium, poor oral intake, and diuretics.^21^ A previous study in the medical emergency department of two tertiary care hospitals showed that psychiatric disorders were common comorbidities in primary polydipsia (65%).^22^ Our results thus add to the understanding of the cause of hyponatremia in psychiatric patients.

It can be difficult to diagnose hyponatremia and it may require a thorough investigation. An unknown etiology is not uncommon. The rates of laboratory tests and an unknown cause in our study were comparable with those of previous studies. A study in the UK showed that paired serum and urine osmolality and sodium were measured in only 23% of participants and that patients with an unknown cause accounted for 58%.^21^ A single‐center study in Korea found that in 14.7% of patients with severe hyponatremia, the exact causes could not be determined due to incomplete laboratory studies.^23^

Our study has several limitations. First, because of its retrospective design, there are missing variables. We could not investigate the symptoms on admission because we did not use the specific questionnaire. We determined the main cause of hospitalization retrospectively, but it might be different from the decision made by physician in charge at admission. We could not determine the cause of hyponatremia because the diagnosis was not always specified in the patient record or because the laboratory testing was insufficient. In particular, the etiology was unknown in one‐third of the nonpsychiatric patients. However, the proportions of insufficient examinations and unknown cases were not larger than in previous studies.^21^, ^23^ Second, we divided hyponatremia cases into psychiatric patients and nonpsychiatric patients. Although the disease profiles of schizophrenia and depression were different, we could not perform subgroup analysis because of the small sample size. Third, because of the small sample size and low mortality rate, we could not elucidate statistically significant risk factors for death.

Despite these limitations, this study provides additional evidence, particularly from patients with psychiatric diseases. Although hyponatremia in psychiatric patients differs from that in the general population and is clinically important, few studies have investigated this issue. Future well‐designed studies with sufficient cases and data are therefore necessary. Our data showed that patients with hyponatremia were often hospitalized for diagnosis other than hyponatremia. This implies that hyponatremia can be found incidentally by blood test. It is important to monitor the serum sodium regularly because hyponatremia in psychiatric patients is not rare. Physicians should consider the possibility of hyponatremia especially when psychiatric patients complaint of malaise or appetite loss because these symptoms can be caused by not only psychiatric diseases but also hyponatremia.

## AUTHOR CONTRIBUTION

Akahito Sako is the guarantor of this work and had full access to all of the data in the study and takes responsibility for the integrity of the data and the accuracy of the data analysis. Study concept and design: Akahito Sako. Acquisition, analysis, or interpretation of data: Takahide Hashimoto, Eriko Makino, Akahito Sako. Drafting of the manuscript: Akahito Sako. Critical revision of the manuscript for important intellectual content: Eriko Makino, Takahide Hashimoto, Hideki Nanasawa, Tetsuro Enomoto, Tatsuro Hayakawa, Hidetaka Hamasaki, Hidekatsu Yanai. Statistical analysis: Takahide Hashimoto, Eriko Makino, Akahito Sako. Administrative, technical, or material support: Hidekatsu Yanai. Study supervision: Hidekatsu Yanai. All authors approved the final version of the manuscript.

## CONFLICT OF INTEREST STATEMENT

No potential conflicts of interest relevant to this article were reported.

## ETHICS APPROVAL STATEMENT

The institutional review board approved the study protocol (NCGM‐G‐001911).

## PATIENT CONSENT STATEMENT

Based on the ethical guidelines, an opt‐out consent method was chosen.

## CLINICAL TRIAL REGISTRATION

This study was not registered.

## Data Availability

The datasets generated during and/or analyzed during the current study are not publicly available because this is a single center study investigating patient medical records, but they are available from the corresponding author on reasonable request.
